# Serum Metal Ion Concentrations in Paediatric Patients following Total Knee Arthroplasty Using Megaprostheses

**DOI:** 10.1155/2014/817257

**Published:** 2014-09-08

**Authors:** Jörg Friesenbichler, Patrick Sadoghi, Werner Maurer-Ertl, Joanna Szkandera, Mathias Glehr, Kathrin Ogris, Matthias Wolf, Christian Weger, Andreas Leithner

**Affiliations:** ^1^Department of Orthopaedic Surgery, Medical University of Graz, Auenbruggerplatz 5, 8036 Graz, Austria; ^2^Department of Oncology, University Clinic of Internal Medicine, Medical University of Graz, Austria; ^3^Institute for Forensic Medicine, Medical University of Graz, Austria

## Abstract

The purpose of this study was to determine the concentrations of cobalt, chromium, and molybdenum in the serum of paediatric tumour patients after fixed hinge total knee arthroplasty. Further, these metal ion levels were compared with serum metal ion levels of patients with other orthopaedic devices such as hip and knee prostheses with metal-on-metal or metal-on-polyethylene articulation to find differences between anatomical locations, abrasion characteristics, and bearing surfaces. After an average follow-up of 108 months (range: 67 to 163) of 11 paediatric patients with fixed hinge total knee arthroplasty, the mean concentrations for Co and Cr were significantly increased while Mo was within the limits compared to the upper values from the reference laboratory. Furthermore, these serum concentrations were significantly higher compared to patients with a standard rotating hinge device (*P* = 0.002 and *P* < 0.001) and preoperative controls (*P* < 0.001). On the other hand, the serum levels of patients following MoM THA or rotating hinge arthroplasty using megaprostheses were higher. Therefore, periodic long-term follow-ups are recommended due to the rising concerns about systemic metal ion exposure in the literature. Upon the occurrence of adverse reactions to metal debris the revision of the fixed hinge implant should be considered.

## 1. Introduction

The potential harmful effects of elevated systemic exposure to cobalt (Co), chromium (Cr), and molybdenum (Mo) are of increasing concern in the literature, especially following metal-on-metal (MoM) hip resurfacing or large diameter MoM total hip arthroplasty (THA) [1]. Bearing surfaces of implants might wear and release particles depending on the tribological pairing. Metal-on-metal articulations were introduced in an attempt to reduce this wear debris. Nevertheless, against all expectations, the short-, mid-, and long-term results of metal ion measurements following MoM THA as well as resurfacing arthroplasty revealed elevated levels of these metals in blood and urine, which represents the systemic exposure [1–9]. Further, it has been shown that high metal ion concentrations are toxic and known to interfere with biological functions [10–14].

Several studies showed serious local adverse reactions against metal debris (ARMD) such as delayed hypersensitivity reactions (ALVAL), osteolysis, pseudotumour formation, metallosis, and local soft tissue reactions such as inflammation and necrosis [1–5, 15–17]. Furthermore, there are three cases of Co intoxication following THA reported in the literature [6, 7, 9]. Overall, the number of revision surgeries due to high metal ion concentrations is still rising, especially following the recall of the ASR device (ASR XL Head and ASR resurfacing device, DePuy, Warsaw, IN).

On the other hand, there is only little data published concerning metal ion concentrations following reconstructions at other anatomical locations than the hip such as the spine or the knee joint. There are two studies in the literature reporting metal ion concentrations following total knee replacement [18, 19]. Further, Zeh et al. [20, 21] and Bisseling et al. [22] related metal ion concentrations following MoM total disc replacement (TDR).

Due to the current debate in the literature about potential disadvantages of MoM articulations, we decided to perform this study to evaluate if there is also a high metal ion release following total knee arthroplasty using fixed hinge megaprostheses. The aim was to determine the serum metal ion concentrations in paediatric patients following fixed hinge total knee arthroplasty after wide tumour resection. Furthermore, these concentrations were compared with serum metal ion levels of patients with other orthopaedic devices such as hip and knee prostheses with metal-on-metal or metal-on-polyethylene articulation. Therefore, the Co and Cr concentrations of patients with MoM total hip replacements were used from an ongoing trial as well as their preoperative metal ion levels. We also compared Co and Cr levels from patients with a standard rotating hinge device and patients with rotating hinge megaprostheses.

The hypothesis of the study was that serum metal ion levels in paediatric patients were higher than in patients with MoM THA or in patients with rotating hinge devices due to the fixed hinge metal-on-metal articulation pairing.

## 2. Patients and Methods

### 2.1. Study Population

**Figure 1:**
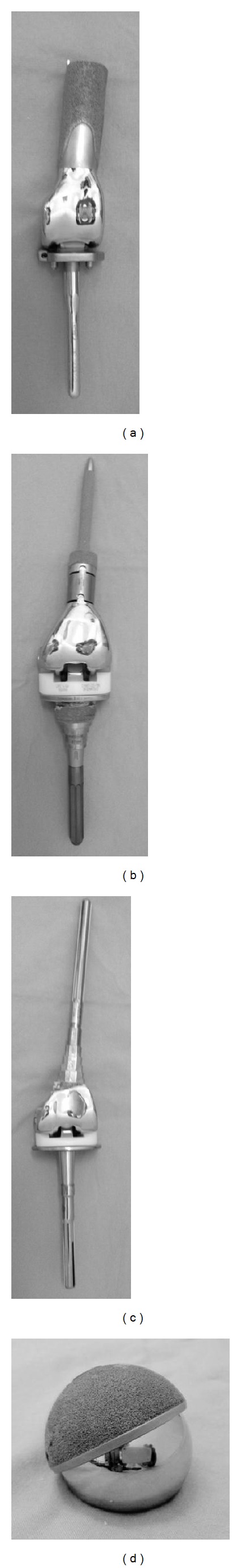
The different devices under investigation: (a) HMRS, (b) LPS, (c) S-ROM Noiles, and (d) ASR XL Head.

From May 1998 to April 2006, 19 paediatric patients (12 male and 7 female) underwent total knee replacement using the fixed hinge Howmedica Modular Resection System (HMRS, Stryker Howmedica Osteonics, Rutherford, NJ; Figure 1(a)) following wide tumour resection around the knee. There were 14 distal femoral, four proximal tibial, and one total femoral replacement for 13 osteosarcomas and six Ewing sarcomas. All patients got neoadjuvant and adjuvant chemotherapy according to the corresponding protocol (COSS/EURAMOS, EUROWING). The mean age at operation was 14 years (range: 9 to 23) and the mean resection length was 20 centimeters (range: 10 to 45). All prostheses were manufactured from a cobalt-chrome-molybdenum alloy according to ISO 2007-4-211.

At time of evaluation, out of these 19 patients, three had died due to their underlying disease and three patients were lost to follow-up. Two patients did not want to participate in our investigation. Overall, 11 patients with a mean follow-up of 108 months (range: 67 to 163) were available for the current study.

### 2.2. Control Groups

#### 2.2.1. Rotating Hinge Knee Groups (RHK)

The characteristics of these patients have been described in a previous study [18]. There were 17 megaprostheses (Limb Preservation System; LPS/M.B.T., DePuy; Figure 1(b)) and eight standard rotating hinge devices (S-ROM Noiles, DePuy; Figure 1(c)). The mean follow-up was 35 months (range: 9–67 months) and all prostheses were manufactured from an ISO-certificated cobalt-chrome-molybdenum alloy (ISO 5832-4).

#### 2.2.2. Total Hip Arthroplasty Group (THA)

Thirty-two patients underwent metal-on-metal large diameter total hip arthroplasty between March 2007 and July 2008 (ASR XL Head, DePuy; Figure 1(d)). Patients' characteristics have been described in a previous study [23]. The prosthesis was manufactured from Co-Cr-Mo alloy according to ISO 5832-4.

For this study, the 12-month data and the 24-month data were regarded as controls because metal ion levels are known to be increased during a running-in period of approximately 6 to 24 months, after which they stabilize. Furthermore, the preoperative baseline metal ion concentrations of these 32 patients were used as controls.

### 2.3. Blood Collection and Serum Metal Ion Analysis

Blood was taken using stainless-steel needles attached to no additive plastic vacuum tubes (VACUETTE, Greiner Bio-One GmbH, Kremsmünster, Austria). All needles and tubes were from the same batch. None of the patients had a history of renal impairment. All specimens were centrifuged at 4000 rpm within 2 hours and stored at −10°C until analysis. The concentrations of Co, Cr, and Mo were determined using electrothermal graphite furnace atomic absorption spectrometry (ET ASS) in an external laboratory (Medizinische und chemische Labordiagnostik Lorenz & Petek GmbH, Graz, Austria). The levels of metal ions in the serum were recorded in concentrations expressed as *μ*g/L. The results were analysed in order to calculate the mean level of each ion in the plasma and the detection limits were 0 to 0.5 *μ*g/L for Co, 0 to 1.9 *μ*g/L for Cr, and 0 to 1.0 *μ*g/L for Mo.

### 2.4. Statistical Analysis

The collected data was processed for statistical differences between the different implant groups. Due to the asymmetric distribution of all parameters nonparametric tests (*Kruskal-Wallis test, Mann-Whitney U test*) were used. Additionally, the correlation between the metal ions was determined using the Pearson correlation coefficient. A *P* value of <0.05 was considered to be statistically significant. For statistical analysis the PASW Statistics 16.0 program (SPSS Inc., Chicago, IL) was used.

## 3. Results

**Figure 2:**
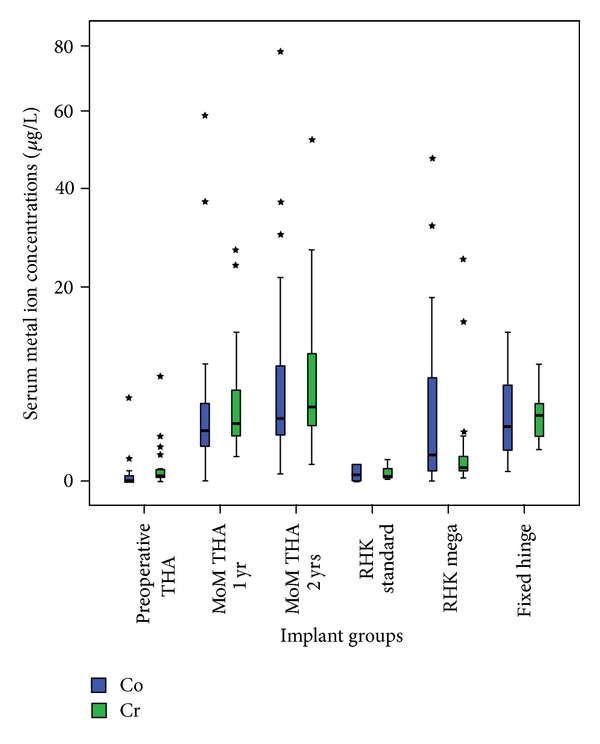
Box plot showing the cobalt and chromium serum metal ion levels in the fixed hinge megaprosthesis group in proportion to the other prostheses and the control group. Patients with fixed hinge devices had higher concentrations of Co and Cr in the plasma compared to preoperative controls and patients with a standard rotating hinge device. These differences reached a statistically significant difference (Table 1).

The characteristics and results of all implant groups are presented in Table 1 and Figure 2.

### 3.1. Study Population

The concentrations of Co, Cr, and Mo in the serum of paediatric patients with fixed hinge megaprostheses were 4.7 *μ*g/L (range: 0.4–12.8 *μ*g/L), 4.01 *μ*g/L (range: 1.48–8.91 *μ*g/L), and 0.6 *μ*g/L (range: 0.1–0.9 *μ*g/L). Compared with the upper limits of the reference values from the laboratory, the values for Co (normal range: 0–0.5 *μ*g/L) and Cr (normal range: 0–1.9 *μ*g/L) were increased ninefold and twofold, respectively, while Mo (normal range: 0–1.0 *μ*g/L) was within the physiological limits. Nevertheless, the average concentrations of Co and Cr were within the internationally accepted limits of 2.0 *μ*g/L to 7.0 *μ*g/L at which a regular follow-up is recommended [24, 25].

Statistical analysis showed that there was no correlation between implant length, follow-up, and serum metal ion concentrations neither for Co nor for Cr (Table 1).

### 3.2. Fixed Hinge Megaprostheses versus Control Groups

The serum concentrations of Co and Cr in the paediatric patients following fixed hinge total knee arthroplasty were significantly higher compared to the preoperative THA controls (Co and Cr: *P* < 0.001, Mann-Whitney  *U* test) and the group with the standard rotating hinge device (Co and Cr: *P* = 0.002 and *P* < 0.001, Mann-Whitney  *U* test, Table 1). On the other hand, the serum metal ion levels of patients following MoM THA were higher at one and two years of follow-up likened to the fixed hinge group, although these differences were not statistically significant (Table 1).

Interestingly, the serum concentrations of Co were higher in the patient group with the rotating hinge megaprosthesis, while the Cr values in this group were lower compared to the paediatric patients with the fixed hinge prosthesis. The difference between the Co concentrations was not statistically significant (*P* = 0.312) while the Cr levels were significantly higher in the fixed hinge group (*P* = 0.002, Mann-Whitney *U* test, Table 1).

There was a positively significant correlation between serum Co and Cr concentrations in all implant groups under investigation (Pearson correlation coefficient, Table 1).

## 4. Discussion

The current study revealed increased serum levels for Co and Cr in paediatric patients following fixed hinge total knee arthroplasty using megaprostheses in comparison to several other implant groups as well as the preoperative controls (Table 1). As expected, referring to previous published data, the values for Mo were within the limits [4, 18, 26–29]. On the other hand, patients with rotating hinge megaprostheses and patients with MoM THA revealed higher serum metal ion concentrations compared to the fixed hinge megaprostheses group although these differences were not statistically significant.

Nevertheless, the hypothesis of this study was not supported by the current results. However, continued long-term follow-up is strictly recommended because especially young patients might suffer from possible late effects of chronic, high systemic metal ion exposure with unknown pathologic effects.

Regarding the official guidelines of the EFORT and AAOS societies for MoM THA, the concentrations of Co and Cr were within the accepted limits of 2.0 *μ*g/L to 7.0 *μ*g/L at which a regular follow-up is recommended [24, 25], although there are no standards defined for other orthopaedic devices than the hip. Nonetheless, the authors believe that these guidelines should also be accepted for other orthopaedic devices, especially with metal-on-metal articulation, to perform continued follow-ups with metal ion determination due to the potential harmful effects of high Co and Cr levels. Further, the systemic toxicity of high Co and Cr levels is always the same and is independent of the type of device.

One limitation of the study is the absence of preoperative concentrations of Co and Cr in the serum of patients with fixed hinge and rotating hinge knee prostheses. Another limitation was that only a small number of patients were enrolled in the study but it was not possible to include further patients because the device under investigation has been pulled from the market several years ago. Furthermore, it should be noticed that there are differences between the follow-ups of the different implant groups. Patients with fixed hinge had the longest follow-up but we do not think that this difference is a major confounding factor because all patients have passed the running-in phase of the implant and the metal ion levels must have stabilized. In addition, statistical analysis did not show any correlation between time of follow-up and serum metal ion concentrations in the different groups.

On the other hand, it should be noted as significant benefit that this is the first study evaluating the increase of serum metal ion levels in paediatric patients following fixed hinge total knee arthroplasty using megaprostheses. Further, these results were compared to the concentrations of different other implant groups. All samples of each group were evaluated at the same laboratory using the same study protocol.

An explanation for the increased concentrations of Co and Cr might be corrosion of the implants, which is known to be proportional to the surface area of the components, and abrasive wear of the soft tissues. Furthermore, metal ions might also be released from the conical junctions of the modular parts from the implants due to fretting.

Our data also shows that the implant's size (mean resection length: 20 cm) might also play an important role, because patients with megaprostheses (fixed hinge and rotating hinge) showed higher concentrations of Co and Cr than patients with a standard rotating hinge device, although there was no correlation between serum metal ion levels and implant size [18].

**Figure 3:**
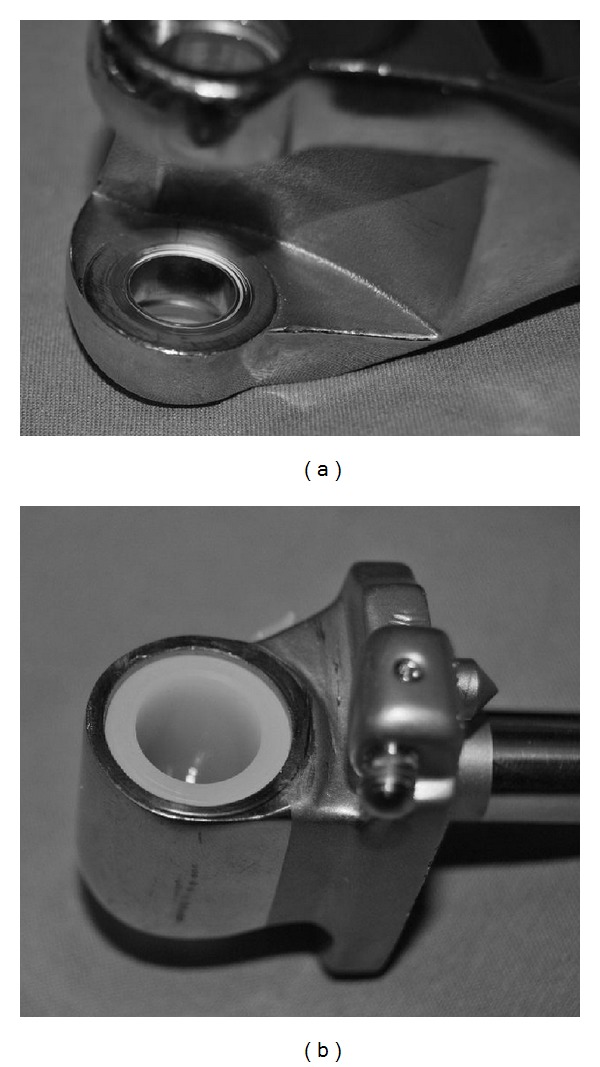
(a) The femoral and (b) the tibial component of an explanted HMRS megaprosthesis. At the side of the hinge there is a direct metal-on-metal articulation clearly with signs of metal debris (scratches and striation).

Another reason for increments of Co and Cr in the fixed hinge group might also be the abrasive wear of the polyethylene bushes at the side of the hinge axle and the direct metal-on-metal contact (Figures 3(a) and 3(b)). Nevertheless, we are unable to provide a safe range of Co and Cr concentrations in the serum because of the wide variation of levels encountered and, therefore, it can be stated that there is a long-term ion release following fixed hinge total knee arthroplasty as observed after rotating hinge total knee arthroplasty [18].

Local soft tissue reactions, delayed hypersensitivity reactions, or development of soft tissue formations like pseudotumours as results of metal ion debris seems to be unlikely following total knee arthroplasty using megaprostheses neither with fixed hinge nor with rotating hinge articulation. Nevertheless, there are two cases of soft tissue masses posterior to the implant following MoM TDR and one case of a wear debris induced pseudotumour seven years following TKA reported in the literature [30–32].

**Figure 4:**
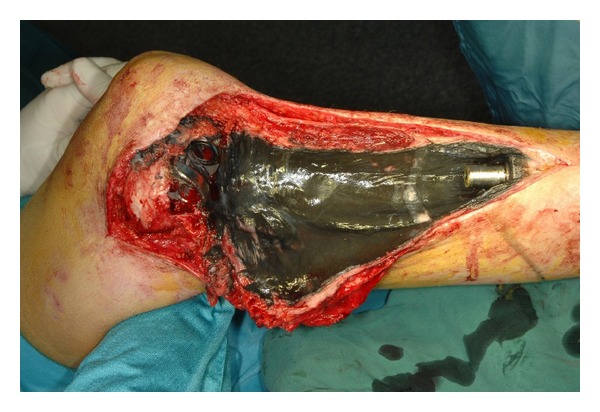
Intraoperative photograph at revision of a fixed hinge megaprosthesis in a 20-year-old male patient showing a marked metallosis in the periprosthetic soft tissues of the lower leg 119 months after implantation.

Another observation we made at revision of fixed hinge megaprostheses was periprosthetic metallosis, whereas metal debris can be found in the joint fluid as well as the surrounding soft tissues including the synovial layer and joint capsule (Figure 4).

Significant factors associated with metal wear are patient's activity, weight, type of articulation (constrained versus nonconstrained), implant's design and geometry, bearing surfaces, alignment, and inlay's quality as well as high contact stresses. Furthermore, corrosion of the implant might play another important role for metallosis [32–34] Romesburg et al. [35] related that titanium components seem to have an increased association with metallosis when compared to Co-Cr implants [36]. On the other hand, Willis-Owen et al. [37] reported early metallosis after TKA in 15 Co-Cr knees needing revision of the implant combined with complete synovectomy. In this series, the complications were associated with a failure in manufacturing of the implant.

## 5. Conclusion

To the author's best knowledge, this is the first study reporting serum metal ion concentrations in paediatric patients and young adults following limb salvage surgery using orthopaedic megaprostheses.

1. Determination of serum metal ion concentrations revealed significant increments for Co and Cr following fixed hinge total knee arthroplasty using megaprostheses. The measurement values were comparable to the results of rotating hinge megaprostheses despite the different articulation mechanisms. Therefore, we believe that there should be a cause of concern due to long-term exposure to Co and Cr when using this type of prosthesis.
2. Further, periprosthetic metallosis was observed at revision surgery which might cause problems like osteolysis and aseptic loosening. On the other hand, there was no correlation between implant size and serum metal ion levels, probably brought about by the small number of patients enrolled in the current series. Nevertheless, serum metal ion levels might be used as indicator for periprosthetic metallosis.
3. Upon the occurrence of adverse reactions to metal debris or intoxications, independently of serum metal ion concentrations, the revision of the fixed hinge implant to the rotating hinge device or another reconstruction method should be considered.

## Figures and Tables

**Table 1:** Demographic data of the study population and mean metal ion levels in *μ*g/L (range) in the different prosthesis groups. The *P* value indicates differences between several implant groups compared to the fixed hinge group. Furthermore, correlations between Co and Cr levels in the implant groups as well as metal ion concentrations and follow-up are shown (THA: total hip arthroplasty; RHK: rotating hinge knee arthroplasty; yr/yrs: year/years; mths: months; FU: follow-up).

	Fixed hinge prostheses (*n* = 11)	Preoperative THA (*n* = 32)	MoM THA 1 yr (*n* = 32)	MoM THA 2 yrs (*n* = 32)	Standard RHK (*n* = 8)	Megaprostheses RHK (*n* = 17)
Mean age (yrs)	14 (10 to 23)	52 (40 to 61)	n.a.	n.a.	73 (60 to 81)	49 (15 to 83)
Sex ratio (m : f)	9 : 2	17 : 15	17 : 15	17 : 15	4 : 4	12 : 5
Mean follow-up (mths)	108 (67 to 163)	n.a.	12 (12 to 12)	24 (24 to 24)	37 (15 to 62)	34 (9 to 67)
Co serum (*μ*g/L)	4.7 (0.4 to 12.8)	0.3 (0 to 5.4)	6.0 (0 to 58.5)	9.3 (0.3 to 78.2)	0.3 (0 to 0.7)	7.5 (0 to 47.0)
Cr serum (*μ*g/L)	4.01 (1.48 to 8.91)	0.68 (0.07 to 7.53)	5.86 (1.11 to 26.56)	8.31 (0.71 to 51.98)	0.33 (0.06 to 0.95)	2.98 (0.12 to 24.90)
*P* value Co/Cr	—	<0.001/<0.001	0.852/0.731	0.501/0.235	0.002/<0.001	0.312/0.002
Correlation Co : Cr	0.742 (*P* = 0.009)	0.945 (*P* < 0.001)	0.869 (*P* < 0.001)	0.967 (*P* < 0.001)	0.764 (*P* = 0.027)	0.945 (*P* < 0.001)
Correlation Co : FU	0.240 (*P* = 0.476)	x	x	x	0.099 (*P* = 0.816)	0.300 (*P* = 0.242)
Correlation Cr : FU	0.137 (*P* = 0.688)	x	x	x	0.007 (*P* = 0.987)	0.373 (*P* = 0.140)
